# Oxidative Stress in Asthma Pathogenesis: Mechanistic Insights and Emerging Biomarker Signatures

**DOI:** 10.3390/ijms27083376

**Published:** 2026-04-09

**Authors:** Justina B. Semyte, Violeta Kvedariene

**Affiliations:** 1 Faculty of Medicine, Vilnius University, LT-03101 Vilnius, Lithuania; justina.semyte@mf.stud.vu.lt; 2 Institute of Clinical Medicine, Clinic of Chest Diseases, Immunology and Allergology, Faculty of Medicine, Vilnius University, LT-03101 Vilnius, Lithuania; 3 Department of Pathology and Forensic Medicine, Institute of Biomedical Sciences, Faculty of Medicine, Vilnius University, LT-03101 Vilnius, Lithuania

**Keywords:** oxidative stress, asthma, biomarkers

## Abstract

Bronchial asthma is a heterogeneous disease characterized by chronic airway inflammation. Oxidative stress arises when the production of free radicals exceeds the antioxidant defense system’s capacity, leading to a redox imbalance. Under oxidative stress, airway inflammation is activated, leading to airway remodeling and maintenance of bronchial hyperreactivity. Airway epithelial remodeling can cause irreversible tissue fibrosis in asthma patients, thereby contributing to a severe course of asthma. A comprehensive literature review was performed using medical database “PubMed” and specialized search engine “Google Scholar” using the PICO model. A total of 51 scientific studies published in English from 2020–2025 were analyzed. Out of the initial 561 articles, 510 articles were excluded due to incomplete articles, studies involving animals, or articles not in English. New studies show that oxidative stress can be objectively measured using various biomarkers. This research aims to provide a better understanding of how oxidative stress affects the airways of asthma patients and what information can be obtained by measuring oxidative stress biomarkers.

## 1. Introduction

Bronchial asthma is a heterogeneous disease characterized by chronic airway inflammation. This condition manifests with varying degrees of shortness of breath, wheezing, coughing attacks, and chest tightness. Asthma symptoms and their intensity change over time, accompanied by variable airway obstruction [1]. This disease differs in prevalence and clinical severity across different parts of the world, yet it remains one of the most common chronic diseases, with increasing incidence and mortality [2]. Clinical asthma exacerbations lead to more frequent hospitalizations, reduced work capacity, increased need for emergency care, and higher mortality. Symptoms typically appear at an early age, and the disease persists throughout life. It impairs lung function and can eventually lead to permanent irreversible bronchial obstruction [3,4]. Bronchial asthma can be provoked by a range of modifiable risk factors, such as allergens, infections, tobacco smoke, intense physical exertion, and cold air, as well as by individual patient characteristics, including hormonal influences, obesity, genetic predisposition, and systemic eosinophilia. The interplay of these factors drives chronic airway inflammation and promotes the pathological processes characteristic of bronchial asthma, including airway hyperreactivity, remodeling, and obstruction. [4]. Overproduction of free radicals damages the airway epithelium, increases mucus hypersecretion, promotes infiltration of immune cells in the airways, and triggers airway hyperresponsiveness and remodeling [5]. Antioxidants, by neutralizing free radical-induced damage, suppress airway inflammation and other processes associated with oxidative stress (OS) [6]. Bronchial asthma is a widespread chronic disease that requires long-term treatment. Medications are prescribed to suppress inflammation in the bronchial tissue, control symptoms, and reduce the frequency of exacerbations. However, the use of medications might also provoke oxidative stress and influence the pathogenesis of asthma [7,8,9]. In addition, the effect of OS can be objectively measured using biomarkers in asthma patients [10]. This research aims to provide a better understanding of such markers in asthma diagnosis and their potential use in clinical practice.

## 2. Methodology

This literature review was carried out using the medical database “PubMed“ (Medline) and a specialized search engine, “Google Scholar”. The literature search was guided by the PICO framework. The population of interest consisted of individuals with asthma. The intervention examined across the publications was oxidative stress, with comparisons drawn between the effects of oxidative stress on asthmatic individuals and on healthy airways. The primary outcome focused on how oxidative stress influences asthmatic airways. Keywords and combinations were used, including but not limited to terms such as: “bronchial asthma,” “asthma,” “oxidative stress,” “oxidative damage,” “oxidative injury,” and “reactive oxygen species”.

### 2.1. Inclusion Criteria

- Scientific literature on subjects with asthma.
- Scientific literature presenting the impact of oxidative stress on the course of asthma.
- Scientific studies on oxidative stress biomarkers in relation to clinical aspects of asthma.
- Clinical literature published in English.
- Scientific literature published no earlier than 5 years ago.

### 2.2. Exclusion Criteria

- Clinical studies on other diseases influenced by oxidative stress (chronic obstructive pulmonary disease, obesity, metabolic syndrome, allergic rhinitis, cardiovascular diseases, pneumonia, atopic dermatitis).
- Experimental studies.
- Studies in which the impact of oxidative stress on the course of asthma is not presented.
- Studies published in languages other than English.
- Animal studies.

### 2.3. Details of Analysis and Interpretation

Using the keywords mentioned above, 561 articles were identified in electronic databases. In the first selection stage, articles whose titles or abstracts met all inclusion criteria were selected. In the second stage, the full texts of the publications were read, and articles that met the exclusion criteria were discarded. The remaining publications were included in the literature review.

### 2.4. Potential Biases and Limitations

During the literature selection process, studies involving other chronic diseases influenced by oxidative stress were excluded. Therefore, the scope of this review is limited to individuals with a confirmed asthma diagnosis and does not include patients with comorbidities such as chronic obstructive pulmonary disease, obesity, metabolic syndrome, allergic rhinitis, cardiovascular diseases, pneumonia, or atopic dermatitis.

Despite the rigorous selection process, there may still be potential biases. The literature review included only open-access studies in English; therefore, it may have excluded some important data written in other languages or studies published in journals with subscription fees. Additionally, the literature included articles that include various methodologies, diagnostic criteria, and biomarkers. As a result, this might have complicated the data synthesis process and affected the understanding of how exactly oxidative stress affects the course of asthma. Future studies are needed to further explore the consequences of oxidative stress in asthma pathogenesis and clinical practice.

## 3. The Effect of Oxidative Stress and Antioxidants on the Respiratory Tract

Oxidative stress is described as an imbalance between increased free radical production and impaired endogenous antioxidant defense systems, with oxidants prevailing [2]. Free radicals, including reactive oxygen species (ROS) and reactive nitrogen species (RNS), are various reactive molecules or atoms that have an unpaired electron in their outer orbital. Because of this property, free radicals easily react with other molecules [11]. In physiological amounts, free radicals are important for regulating protein activity via oxidation–reduction reactions, forming protein structures, and transmitting intracellular signals [12]. However, an excessive amount of radicals, due to their chemical properties, reacts very actively with surrounding molecules, damages the respiratory tract epithelium, increases mucus hypersecretion, promotes immune cell infiltration in the airways, and provokes airway hyperreactivity and remodeling [5].

Oxidative stress can progress when the production of radicals is activated, the body’s antioxidant system is reduced (e.g., low levels of glutathione), antioxidants are not obtained from food (e.g., vitamins), antioxidant enzymes are weakened, or when there is an excessively high activity of pro-oxidant enzymes [13].

Oxidative stress affecting the course of asthma can be classified as endogenous (cellular) or exogenous (external), depending on the source of free radical formation. Endogenous oxidative stress arises when excessive amounts of reactive oxygen and nitrogen species are formed in the human body. In contrast, exogenous oxidative stress occurs when reactive molecules are inhaled into the respiratory tract [3]. Regardless of the source, free radicals are associated with disruptions in cell structure and function—lipid peroxidation, protein modification, and DNA damage [14].

### 3.1. Cellular Sources of Free Radicals and Their Effect on the Lungs

**Figure 1:**
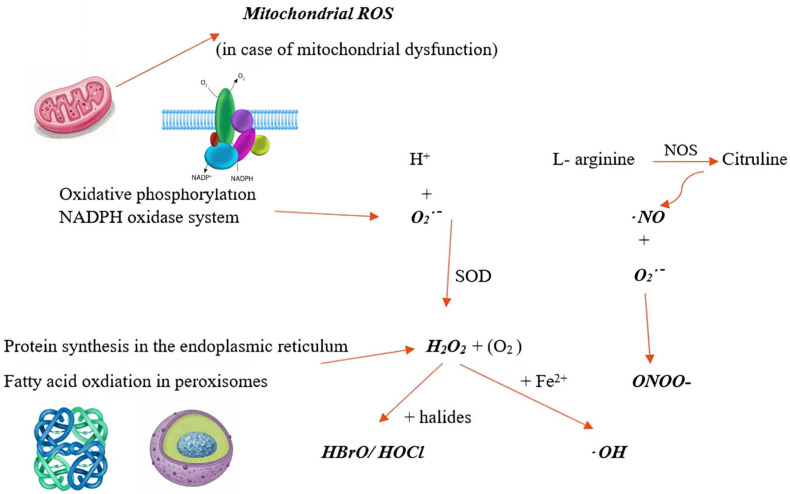
Formation of free radicals in cells (according to [2,3,5,16,17]). In mitochondria, during ATP synthesis or in the NADPH oxidase system, the superoxide anion radical (O_2_^•–^) is produced and reduced to hydrogen peroxide (H_2_O_2_). Hydrogen peroxide also forms during protein synthesis or in peroxisomes during fatty acid oxidation. During the Fenton reaction, Fe^2+^ ions interact with H_2_O_2_ to form the hydroxyl radical (⋅OH). In eosinophils and neutrophils, H_2_O_2_ can react with halides to form reactive molecules—hypobromous acid (HBrO) or hypochlorous acid (HOCl). During the synthesis of amino acid citrulline from L-arginine, the nitric oxide radical (⋅NO) is formed, which reacts with the superoxide anion to form a highly reactive radical—peroxynitrite (ONOO-). In mitochondrial dysfunction, mitochondria-derived ROS are released, damaging the mitochondria. SOD—superoxide dismutase; NOS—nitric oxide synthase; ROS—reactive oxygen species.

ROS are usually produced in the body during cellular respiration, metabolic reactions, immune system function, and protein synthesis. One of the main cellular ROS, the superoxide anion radical (O_2_^•–^), is produced from molecular oxygen during oxidative phosphorylation in mitochondria or by NADPH oxidase systems in immune cells (see Figure 1) [15]. The enzyme superoxide dismutase (SOD) converts the reactive superoxide anion (O_2_^•–^) into a more stable molecule, hydrogen peroxide (H_2_O_2_). Hydrogen peroxide can diffuse into the cytosol and the cell nucleus, activating redox-dependent intracellular signaling pathways [16]. Iron ions (Fe^2+^) react with H_2_O_2_ to form a highly reactive ROS—the hydroxyl radical (⋅OH), which oxidizes and damages various biomolecules (e.g., DNA, proteins, or lipids). This reaction is known as the Fenton reaction [2].

Immune system cells and metabolic processes also contribute to cellular ROS production. The enzymes from eosinophil and neutrophil granules, peroxidases, catalyze the reaction of H_2_O_2_ with halides, leading to the formation of hypobromous and hypochlorous acids, which, in turn, increase oxidative injury and promote the development of asthma. ROS production by immune cells is a major source of oxidative stress in the lungs [3]. Another important ROS, nitric oxide (⋅NO), is produced by the conversion of the amino acid L-arginine to L-citrulline, catalyzed by nitric oxide synthase (NOS). Nitric oxide synthase is a pro-oxidative enzyme with three types: neuronal NOS1, inducible NOS2, and endothelial NOS3 [17]. NOS2 is an enzyme that is most commonly activated by cytokines or pro-inflammatory factors. Under physiological conditions, the ⋅NO radicals formed have a cytostatic effect on cells infected with parasites. Still, in the lungs of asthma patients, they are expressed in higher amounts and, by catalyzing the formation of nitric oxide, damage healthy respiratory tissues [17,18]. Increased ⋅NO levels are associated with severe asthma, and bronchodilator drugs are more effective in such patients. [15] When nitric oxide reacts with the superoxide anion (O_2_^•–^), a highly reactive nitrogen–oxygen radical, peroxynitrite (ONOO-), is formed [16].

Oxidative phosphorylation processes occurring in mitochondria produce ATP molecules and large amounts of free oxygen radicals (e.g., superoxide anions) [19]. In the presence of various external stressors, mitochondrial energy production processes change, and the organelles release molecules (mitochondrial DNA and mitochondrial ROS) that damage their own mitochondria and impair mitochondrial function. Mitochondrial dysfunction is associated with respiratory tract inflammation and hyperreactivity [5]. Other important organelles involved in ROS production are the endoplasmic reticulum, which generates H_2_O_2_ during protein production, and peroxisomes, where fatty acid oxidation and xanthine oxidase-catalyzed processes produce H_2_O_2_ and O_2_^•–^ [16].

### 3.2. External Sources of Free Radicals

External ROS sources may vary and include medications (e.g., chemotherapy), radiation, metal ions, pulmonary circulation disorders (e.g., ischemia–reperfusion-induced injury), and environmental pollution [13]. The most widely studied factors by scientists that cause oxidative stress and influence the course of asthma are air pollutants, which consist of numerous different compounds—particulate matter, ozone, NO, SO_2_, carbon particles, volatile organic compounds, tobacco smoke, and pollution caused by forest fires [20,21,22].

### 3.3. Antioxidants

Cells have protection against excessive oxidation—the antioxidant system neutralizes the effects of oxidants and thus maintains the oxidation–reduction balance in the body. Antioxidants can be classified by their source, mechanism of action, or antioxidant effect [23]. Typically, antioxidants are classified according to their effects as enzymatic or non-enzymatic. The main ones are enzymatic antioxidants that catalyze reactions to convert harmful free radicals into less reactive molecules, and the most important of these are: superoxide dismutase (SOD), glutathione peroxidase (GPx), glutathione reductase (GR), glutathione S-transferase (GST), and catalase (CAT) [24]. SOD catalyzes the conversion of superoxide ions (O_2_^•–^) into H_2_O_2_ and has three isoforms, each requiring a different metal cofactor for activity. Thanks to these enzymes, a less reactive product—hydrogen peroxide—is formed, which the remaining antioxidant enzymes—GPx, CAT—help convert into water through different chemical reactions [11,12].

Non-enzymatic antioxidants protect cells from free radicals by directly reacting with oxidants [15]. These substances can be produced by the body itself or obtained from food [25]. In the respiratory tract, the main non-enzymatic antioxidants are glutathione, vitamins C and E. Other non-enzymatic antioxidants include beta-carotene, flavonoids, procyanidins, bilirubin, and sex hormones (estradiol and estrone) [2,13,25,26].

The glutathione antioxidant system reduces H_2_O_2_ and protects against lipid peroxidation [13]. Glutathione, found in the secretions of the respiratory tract mucosa and in blood plasma, is synthesized by two enzymes: glutathione synthetase or glutamate-cysteine ligase [27]. Catalyzed by GPx, hydrogen peroxide is reduced, forming the oxidized form of glutathione, also known as glutathione disulfide (GSSG). GPx also breaks down lipid peroxidation products that arise when free radicals react with polyunsaturated fatty acids. Reduced glutathione (GSH) and its oxidized form, GSSG, can be transported outside the cell and converted by enzymes into amino acids, which, once inside the cell, are used for further glutathione synthesis [15,16,27,28]. Glutathione metabolism processes are important in regulating the redox balance. Under oxidative stress conditions, cells strive to maintain a constant GSH/GSSG ratio by synthesizing glutathione or reducing GSSG with glutathione reductase [16]. In addition, glutathione S-transferase plays an important role in detoxifying harmful metabolites [29].

Vitamin E, also known as alpha-tocopherol, is a powerful antioxidant that protects the cell membrane from damage by reacting with radicals formed during lipid peroxidation. Although vitamin C also acts as an antioxidant, i.e., it reacts with O_2_^•–^ and OH¯ radicals, it is also a pro-oxidant, meaning it is not a very strong antioxidant [15]. It is known that in individuals with asthma, levels of non-enzymatic antioxidants (vitamin C and E) in the respiratory tract mucosa decrease [2]. Bilirubin, a well-known product of erythrocyte breakdown, is another non-enzymatic antioxidant that, when bound to plasma albumins, can neutralize ROS and protect tissues from lipid peroxidation. It is believed that bilirubin nanoparticle compounds could suppress oxidative processes and reduce respiratory tract inflammation in patients with asthma [26].

## 4. The Impact of Oxidative Stress on the Course of Asthma

### 4.1. Airway Inflammation

Oxidative stress and inflammation are related pathophysiological processes whose mechanisms of action interact and modulate each other. Various external stimuli that enter the lungs damage the respiratory epithelium, trigger an inflammatory response, and promote excessive ROS production in cells. Oxidative stress, in turn, can increase inflammation in the airways and exacerbate the course of asthma [30]. Moreover, immune cells involved in asthma-induced inflammation (neutrophils, eosinophils, macrophages) are the main sources of ROS [31]. Free radicals initiate airway inflammation in the pathogenesis of asthma through three main pathways:

(a) ROS can directly cause oxidation reactions and cell shedding in bronchial epithelial cells of asthma patients. This provokes epithelial cells to release cytokines that activate dendritic cells, which, in turn, cause type 2 innate lymphoid cells and Th2 cells to produce cytokines, thereby contributing to type 2 inflammation.
(b) Oxidative stress stimulates mast cell degranulation and the release of histamine, prostaglandin D2, and other cytokines, thereby increasing mucus hypersecretion and inflammation.
(c) Free radicals indirectly activate transcription factors (e.g., Nuclear factor kappa-light-chain-enhancer of activated B cells (NF-κB) and Activator Protein 1 (AP-1)) [2]. Activation of the NF-κB transcription factor is considered important in regulating both innate and adaptive immune responses in allergic asthma [32].

When excessive free radicals accumulate in the body, Toll-like receptors (TLRs) and the NF-κB pathway are activated [2]. External oxidants, such as ozone or cigarette smoke, promote TLR-stimulated production of pro-inflammatory cytokines and activate the inflammatory response in the lungs [33]. Toll-like receptors are a group of receptors in the respiratory epithelium that recognize pathogen-associated molecular patterns (PAMPs) or damage-associated molecular patterns (DAMPs) and activate immune cells, the production of inflammatory cytokines, and transcription factors, such as NF-κB [32]. TLR-activated transcription factors regulate the gene expression of pro-inflammatory cytokines, enzymes, and other molecules and initiate the inflammatory process [13]. One of the most important transcription factors is NF-κB, which regulates the activity of cyclooxygenase-2 and inducible nitric oxide synthase (NOS2), contributing to the progression of inflammation and the development of OS [34]. Thus, an excess of free radicals affects the receptors and gene expression of the respiratory epithelium, increases airway inflammation, promotes the synthesis of free radicals, and contributes to the progression of asthma.

On the other hand, OS can also activate transcription factors that initiate antioxidant processes. For example, the transcription factor Nrf2 (Nuclear factor 2-related factor) binds to the promoters of genes encoding antioxidant enzymes [35]. Normally, Nrf2 activity in the body is regulated by Kelch-like ECH-associated protein 1 (Keap1). Under mild oxidative stress, the Keap1 protein is inhibited, and the transcription factor Nrf2 translocates to the nucleus and is expressed there, activating the synthesis of antioxidant enzymes. However, under severe oxidative stress, Keap1 function is disrupted, thereby impairing Nrf2 activity and cell antioxidant protection [31]. The Nrf2-regulated response to oxidative stress is important in maintaining the redox balance in lung cells, reducing the harmful effects of oxidants on the lungs, and limiting inflammatory processes [7]. Furthermore, activation of the Nrf2 pathway reduces airway hyperreactivity [3]. Activation of the Nrf2 transcription pathway is a natural cytoprotective system of the body that, in the presence of redox balance, protects against damage caused by free radicals generated from external and cellular influences. In a state of OS, the Nrf2 transcription factor is suppressed, and the antioxidant effect on cells weakens.

In summary, oxidative stress, by acting on cell receptors and transcription factors, activates inflammatory processes that, in turn, promote ROS production. However, OS also affects gene expression and regulates the activity of antioxidant enzymes. The relationship between oxidative stress and immune mechanisms helps us understand that intracellular free radicals and external irritants cause respiratory tract inflammation and regulate protective antioxidant processes.

### 4.2. Airway Remodeling and Hyperreactivity

Airway remodeling involves a series of structural changes in the airways, such as goblet cell metaplasia, fibrosis, airway smooth muscle (ASM) hyperplasia or hypertrophy [36]. Free radicals stimulate the airway epithelium to release the profibrotic cytokine transforming growth factor beta (TGF-β). Along with eosinophils, epithelial cells produce TGF-β, which drives collagen synthesis, fibroblast proliferation, and the activation of other immune cells, thereby contributing to airway remodeling [2]. In this way, chronic inflammation induced by OS ultimately promotes airway remodeling through fibroblast activation, immune cell involvement, and enhanced collagen deposition.

**Figure 2:**
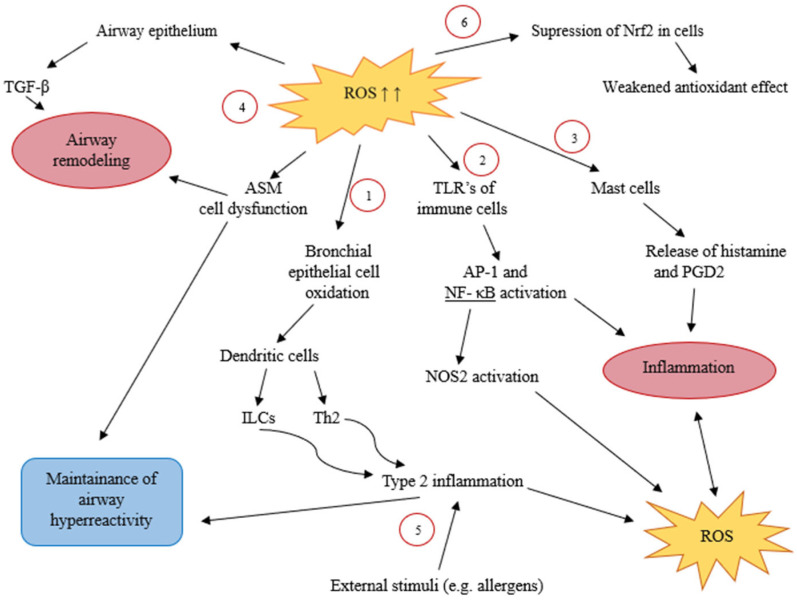
The relationship between oxidative stress and asthma pathogenesis (according to [2,15,31,32,34,37,38]). Oxidative stress affects asthmatic airways by promoting airway inflammation and remodeling, maintaining airway hyperreactivity, and suppressing antioxidant protection. (1) Free radicals can directly cause oxidation reactions in bronchial epithelial cells, as they activate cytokine release that provokes ICLs and Th2 lymphocytes through dendritic cells and contribute to type 2 inflammation; (2) ROS activates transcription factors NF-κB and AP-1 through Toll-like receptors in immune cells, which initiate inflammation. Moreover, the NF-kB pathway regulates the activity of NOS2, which produces free radicals; (3) ROS stimulates mast cell degranulation and the release of histamine, prostaglandin D2, and other cytokines, thereby increasing inflammation; (4) Free radicals stimulate the airway epithelium to release the profibrotic cytokine TGF-β, which drives processes of airway remodeling. In addition, ROS stimulates ASM dysfunction, which enhances airway remodeling and maintains airway hyperreactivity; (5) Both external factors, such as allergens, as well as inflammation caused by OS, trigger type 2 inflammation response that maintains airway hyperresponsiveness and can stimulate ROS production; (6) Excessive free radical formation suppresses Nrf2 transcription factor in cells, resulting in weakened antioxidant protection. ROS—reactive oxygen species, TGF-β—transforming growth factor beta, Nrf2—nuclear factor 2 related factor, ASM—airway smooth muscle, TLR—Toll-like receptor, AP-1—activation protein 1, NF-κB—Nuclear factor kappa-light chain enhancer of activated B cells, PGD2—prostaglandin D2, NOS2—inducible nitric oxide synthase, ILCs—innate lymphoid cells, Th2—T lymphocyte 2 helper cells.

Asthmatic airways are also characterized by airway hyperreactivity, a tendency to respond excessively to environmental stimuli that cause bronchoconstriction [12]. The progression of airway hyperresponsiveness, inflammation, and remodeling is closely linked to dysfunction of the ASM cells. Oxidative stress contributes to ASM dysfunction by promoting excessive secretion of inflammatory mediators, increasing ASM mass, and amplifying inflammatory responses and airway hyperreactivity [37]. Dysfunctional ASM cells proliferate, differentiate, and migrate, further reinforcing airway remodeling, therefore free radicals play an important role in asthma progression. Various external compounds, such as allergens, activate antigen-presenting cells and drive T lymphocyte differentiation, thereby triggering type 2 inflammatory response [38]. The released cytokines maintain airway hyperresponsiveness, enhance pro-oxidant enzyme activity, and stimulate ROS production [15]. In this way, OS impairs ASM function, contributes to airway remodeling, and sustains airway hyperreactivity. Moreover, the inflammatory processes characteristic of asthma contribute to the production of free radicals, reinforcing this pathogenic cycle. The following scheme outlines the mechanism of oxidative stress in the pathogenesis of asthma (see Figure 2).

### 4.3. The Impact of Oxidative Stress on Asthma Treatment

The treatment of bronchial asthma is long-term. Medications for asthma treatment are prescribed to suppress inflammation in the bronchial tree, control symptoms, reduce the frequency of exacerbations, treat asthma-related irreversible airway obstruction, and decrease the risk of death. The most important medications are inhaled glucocorticosteroids (GCSs), usually prescribed as monotherapy or combined with beta-2 adrenergic drugs [1].

It is believed that OS contributes to the development of glucocorticosteroid-resistant asthma. GCs entering the body reduce eosinophilia in the airways, inhibit the production of inducible nitric oxide, and decrease the inflammatory process by binding to glucocorticoid receptors in the cytosol of cells [39]. Models of chronic obstructive pulmonary disease and asthma research suggest that maintaining the redox balance in the body is important for GCs to achieve sufficient drug effect [7]. During oxidative-stress-related asthma exacerbation, there is enhanced activation of NF-κB and AP-1 transcription factor in immune cells. It is thought that excessive activation of NF-κB and AP-1 could be responsible for impaired expression of GC receptors, making it likely that the desired drug effect on the bronchi is not achieved [8]. Based on preclinical studies, it is believed that stimulating endogenous antioxidant systems may improve sensitivity to GCs [7]. OS can alter GC receptor expression and contribute to the development of steroid-resistant asthma.

Beta-2 adrenergic drugs are used to reduce asthma-related bronchospasm, relax bronchial smooth muscles, and alleviate clinical symptoms [40]. A certain amount of free radicals is necessary for beta-2 adrenergic receptor signal transmission. Still, when beta-2 adrenergic drugs enter the lungs, they stimulate the production of ROS, causing oxidative stress. An excess of free radicals likely inhibits the beta-2 adrenergic receptor’s ability to send ROS-dependent signals to cells. Due to receptor inhibition, the signals sent by the receptors decrease, and over time, the drugs’ bronchodilatory effect may weaken [9]. Thus, beta-2 adrenergic drugs intended to reduce asthma symptoms not only improve respiratory function but also provoke oxidative stress. It is believed that OS can inhibit beta-2 adrenergic receptor signal transmission and reduce the bronchodilatory effect, potentially promoting asthma progression.

In summary, the scientific literature examines the interactions between oxidative stress and asthma medications and their effects on clinical outcomes. Excessive levels of free radicals in lung tissue suppress GC receptor expression, while beta-2 adrenergic drugs provoke oxidative stress, thereby eventually impairing receptor signaling. It is believed that oxidative stress contributes to the development of resistance to asthma medications, underscoring the need for further research to clarify the interactions between OS and GCs and beta-2 adrenergic drugs, investigate their impact on the asthma course, and explore the potential of antioxidant therapy.

## 5. Oxidative Stress Biomarkers

Oxidative stress provokes the course of asthma and contributes to airway inflammation, as well as the effects of medications, creating a need to objectively assess the impact of this process on asthma clinical outcomes. The impact of OS on tissues can be evaluated by measuring levels of molecules damaged by free radicals (e.g., proteins, lipids, DNA), assessing oxidative stress status or the capacity of the protective antioxidant system, and examining other molecules associated with the OS process. Summarizing the latest literature, the main types of OS biomarkers used in scientific studies to evaluate the course of asthma are described (see Table 1).

### 5.1. Lipid Peroxidation Products

Malondialdehyde (MDA) is a product of lipid peroxidation and is most commonly used as a marker of oxidative stress [42]. Lipid peroxidation disrupts the integrity of the cell membrane and damages its structure [24]. Typically, in scientific studies, this marker is measured in blood, urine, or exhaled breath condensate to confirm systemic OS, but it can also be detected in nasal secretions. The study results showed that MDA in nasal secretions could be a useful biomarker for monitoring the worsening of asthma in patients associated with air pollution components—particulate matter and ozone [42]. On the other hand, assessing MDA levels in the systemic circulation, along with antioxidant enzyme activity, could be used to predict asthma severity and asthma control [10]. MDA is the most extensively studied oxidative stress biomarker in asthma and can assess the condition of asthma patients. Less frequently studied by researchers, the lipid peroxidation product 8-iso-prostaglandin F2 alpha (8-iso-PGF2α) in blood and sputum is also significantly elevated in individuals with asthma and may indicate systemic oxidative stress [41].

### 5.2. Activity of Enzymatic Antioxidants

Measurement of enzymatic antioxidant activity in blood is another commonly used method for assessing the impact of OS on asthma and determining the capacity of the antioxidant system to protect cells from free radical damage. Scientific studies have shown that the activity of one or more antioxidant enzymes (CAT, GST, GPx, GR) is significantly reduced in patients with asthma, leading to systemic oxidative damage [10,24,29]. However, the activity of the antioxidant enzyme SOD in asthma patients may vary compared to healthy individuals. Clinical studies have shown different results, both decreases [29,43] and increases [10,41] in this enzyme. These changes can be partly explained by differences in the methods used to assess the enzyme, population differences, or individual variations in patients’ antioxidant capacity [10]. It is believed that SOD enzyme activity may increase as a compensatory mechanism in response to oxidative stress, but in people with asthma, this compensatory protection does not exceed the damage caused by OS [41]. Measuring the activity of enzymatic antioxidants, in combination with lipid peroxidation products, may help more accurately assess the course of asthma in patients. Based on clinical trial data, patients with uncontrolled asthma have high levels of free radicals in systemic circulation, and increased MDA levels, SOD activity, and decreased GPx activity were indicators predicting poorly controlled asthma [10].

### 5.3. Amount of Non-Enzymatic Antioxidants

Reduced glutathione, a non-enzymatic antioxidant, is a biomarker of OS and is often measured in studies alongside enzymatic antioxidants. A decreased GSH level promotes lipid peroxidation in tissues, which is characteristic of the oxidative state in asthma [10,28,29]. There can be several reasons for the reduction in glutathione levels in the body: (a) a weakened antioxidant system capacity dominated by oxidants; (b) increased consumption of glutathione in the body; and (c) a deficiency of amino acids needed to form glutathione molecules [28]. All of these reasons lead to disruption of the glutathione antioxidant system, which can also disturb the balance of enzymatic antioxidants.

### 5.4. Metabolites of Oxidized DNA

As is well known, free radicals are highly reactive particles that readily react with surrounding molecules. The oxidized DNA product 8-hydroxy-2-deoxyguanosine (8-OH-dG) is detected in the urine of individuals with asthma and indicates a state of systemic OS in the body [42]. An increase in this biomarker has been observed in children with asthma and is associated with an increase in nearly 27 volatile organic compound metabolites in the child’s body. The results support that asthma progression is triggered by exposure to volatile organic compounds, which induce oxidative stress damage [21].

### 5.5. Biomarkers Characterizing the Overall Oxidative Level or the Antioxidant Capacity in the Body

When investigating blood samples, it is possible to evaluate not only individual molecules affected by OS or components of the antioxidant system, but also their overall ability to oxidize or reduce chemical compounds. The assessment of total oxidant status (TOS) is based on the oxidation of iron ions Fe^2+^. Chemical oxidation reactions occur in an acidic medium, and when trivalent iron Fe^3+^ is formed, a color change in the test sample occurs, which is measured spectrophotometrically and expressed in units of μmol/L. TOS levels can be measured in blood and exhaled breath condensate [24]. Moreover, the body’s oxidative level can be calculated by evaluating derivatives of reactive oxygen metabolites (dROMs) in the blood. The dROMs indicator is used as a marker of oxidative stress, indicating the total amount of hydroperoxides released from proteins, formed during the Fenton reaction when Fe^2+^ ions are oxidized in blood serum [45]. These two investigative methods help assess the excess of oxidants in patients with asthma and to more accurately determine the state of OS. In addition, the body’s antioxidant system can be evaluated. The most commonly used method is the calculation of total antioxidant capacity (TAOC) [14]. This method examines how antioxidants in blood or exhaled breath condensate reduce the free radicals generated. During neutralization, the reagent’s color changes, and antioxidant capacity is assessed by spectrophotometry, with results reported in mmol/L [6,24]. Blood plasma antioxidant capacity can also be evaluated using another method, commonly known as PAT test. It is calculated using photometry, which determines the ability of blood plasma antioxidants to reduce iron ions (Fe^3+^ → Fe^2+^). An elevated PAT level indicates oxidative stress [45]. Furthermore, oxidative stress index (OSI) can be evaluated by calculating the ratio of oxidants to antioxidants [14]. All these indicators characterizing the OS status allow for an overview of the balance between oxidants and antioxidants in asthma patients.

Studies have shown that individuals with asthma exhibit increased TOS and OSI biomarkers compared to healthy individuals. Blood serum antioxidant capacities decrease in individuals with asthma [14], but correlations have been found between the TAOC biomarker and asthma symptom control. Patients with poorly controlled asthma had lower serum TAOC levels than healthy individuals. However, no significant difference was observed between healthy individuals and those with well-controlled asthma in antioxidant capacity. Therefore, it is believed that increased TAOC levels in systemic circulation contribute to good asthma control [6]. However, another study reported that dROM and PAT test values, which assess OS balance, were significantly elevated in those with severe asthma, even though participants’ symptoms were well controlled [45]. Thus, even with adequate symptom control, oxidative stress persists in the bodies of people with asthma; however, it is believed that improving the antioxidant system’s capacity could enhance asthma symptom control.

### 5.6. Reactive Nitrogen Species

Free oxygen radicals can react with nitric oxide to form RNS and provoke inflammation in the respiratory tract [47]. Measuring the amount of molecules associated with nitric oxide, including nitrites (NO_2_-), nitrates (NO_3_-), and total nitric oxide metabolites, allows for the assessment of the amount of unstable ROS. High concentrations of nitrites, nitrates, and total nitric oxide metabolites in the blood serum are associated with uncontrolled asthma [6]. Thus, a high level of RNS molecules provokes inflammation and oxidative stress and contributes to more severe asthma symptoms.

### 5.7. Biomarkers of Thiol–Disulfide Bond Balance

Maintaining the balance of thiol–disulfide bonds is another important mechanism for assessing the state of oxidative stress. Thiols are chemical compounds containing a sulfhydryl (-SH) group and are involved in the formation of free radicals. Oxidation of thiol groups (-SH) leads to disruption of homeostasis and disturbs the balance of the glutathione antioxidant system [48]. Meanwhile, the reduction in thiol–disulfide bonds suppresses OS and maintains the thiol–disulfide bond balance. Therefore, the level of thiol–disulfide bonds, calculated by measuring total thiol content and free thiol content, is a novel marker of oxidative stress, and the assessment of protein -SH groups is a marker reflecting the antioxidant system. Both of these biomarkers are additionally used to evaluate oxidative stress and the clinical manifestations of asthma [14,24].

### 5.8. Biomarkers Associated with Protein Oxidation

Free radicals, by oxidizing proteins, generate various molecules, including oxidized protein hydroperoxides and advanced oxidation protein products (AOPPs), which can be used as OS biomarkers [43]. Protein hydroperoxides are early, unstable compounds formed when free radicals react with proteins, and measuring them requires advanced technologies, such as the fluorescent coumarin boronic acid assay [44]. AOPPs are formed as terminal compounds when free radicals react with proteins, and they have already lost their oxidative potential [49]. Protein oxidation leads to oxidative damage to DNA, so the overall protein oxidation index is another biomarker indicating the general oxidative damage to cells and the organism’s adaptive capacity [43]. The increase in protein oxidation index and AOPPs is associated with uncontrolled asthma, while the protein hydroperoxide biomarker is associated with airway inflammation. Therefore, it is believed that proper asthma control could reduce OS [10,43,44].

### 5.9. Other Biomarkers

New biomarkers of oxidative stress and inflammation are being studied not only for asthma control but also for identifying severe asthma. Mitochondrial dysfunction contributes to OS, so by comparing the amount of mitochondrial DNA (mtDNA) with nuclear DNA (nDNA), it is possible to assess the state of oxidative stress in asthma patients from blood or exhaled breath condensate samples [50]. A recent scientific study showed that using quantitative real-time PCR, the mtDNA/nDNA ratio can be calculated. According to the study data, this ratio was significantly higher in individuals with severe asthma compared to those with moderate or mild asthma. The mtDNA/nDNA biomarker could also be useful for evaluating a specific asthma endotype, since the highest mtDNA concentration was observed in the Th2-independent severe asthma endotype [46]. Further studies are needed to confirm the impact of mtDNA and nDNA biomarkers on OS processes and asthma progression.

Ceruloplasmin, a copper-binding acute-phase protein, is involved in the inflammatory process and serves as an additional biomarker of the antioxidant system. This protein is important for inhibiting the pro-oxidative enzyme metalloproteinase in neutrophils, catalyzing the oxidation of Fe^2+^, and may suppress the effects of free radicals [51]. Ceruloplasmin is measured in combination with other biomarkers and can help assess the impact of OS in asthma patients. It has been observed that MDA and ceruloplasmin levels are elevated in patients with non-allergic asthma compared with those with allergic asthma [24].

### 5.10. Potential Benefits of Biomarkers in Clinical Practice

Assessment of OS biomarkers could provide specialist doctors with more accurate information about systemic oxidative stress, asthma symptom control, and disease severity and help clarify the asthma endotype. Measuring individual or combined OS biomarkers provides useful data (see Table 2). All the biomarkers discussed allow the detection of an increased oxidative stress state. However, the overall balance of oxidative stress in the body is more broadly assessed using TOS, TAOC, PAT, d-ROMs, and the oxidative stress index. The level of asthma control can be determined by measuring MDA along with enzymatic antioxidants, TAOC, the concentrations of nitrites, nitrates, and total nitric oxide metabolites in blood serum, the protein oxidation index, and AOPPs.

For the assessment of severe asthma clinics, it may be potentially useful to calculate the mtDNA/nDNA ratio and perform dROMs and PAT tests, and nasal secretion MDA, as well as urinary 8-OHdG biomarkers, could help monitor the deterioration in the condition of asthma patients associated with air pollution components. According to new research data, OS biomarkers (e.g., MDA, ceruloplasmin indicators, mtDNA concentration) may even be associated with certain types of asthma and contribute to the evaluation of the immunological profile of asthma. Due to the high variability of OS biomarkers, further studies are needed to assess the applicability in clinical practice and to confirm the association of existing oxidative stress biomarkers with asthma in a broader patient population.

## 6. Conclusions and Future Directions

New scientific studies examining the pathogenesis of asthma focus on the role of oxidative stress, the formation of free oxygen radicals, and the effects of antioxidants on asthma progression. The effects of free oxygen radicals on the lungs play both physiological and pathological roles in human body cell functions, affect intracellular signaling pathways and the activity of antioxidant enzymes, and cause damage to DNA, proteins, lipids, and other molecules. On the other hand, oxidative stress provokes pathophysiological mechanisms characteristic of asthma—airway inflammation, hyperreactivity, and remodeling. There is evidence that OS contributes to the development of resistance to asthma medications; therefore, further scientific studies are needed to clarify the interactions between oxidative stress and glucocorticosteroids and beta-2 adrenergic drugs, to investigate their impact on the course of asthma, and to explore the effect of oxidative stress on other medications such as leukotriene receptor antagonists or monoclonial antibodies.

Assessing OS biomarkers could help clinicians better understand oxidative status in asthma patients, as it can be related to disease severity, symptom control, and the Th2-independent severe asthma endotype. The main studied biomarkers are malondialdehyde and enzymatic antioxidants (e.g., superoxide dismutase, catalase, glutathione peroxidase). Still, many new oxidative stress biomarkers are emerging—biomarkers associated with protein oxidation, thiol–disulfide balance, the ratio of mitochondrial DNA to nuclear DNA, reactive oxygen metabolite derivatives, and tests of blood plasma antioxidant capacity, among others. Given the wide variety of oxidative stress biomarkers, further research is needed to confirm their relationship with asthma in larger patient populations and to evaluate their applicability in clinical practice.

## Figures and Tables

**Table 1:** Biomarkers reflecting oxidative stress, relevant for the assessment of bronchial asthma.

Type of OS Biomarkers	Biomarkers
Lipid peroxidation products	8-iso-prostaglandin F2 alpha (8-iso-PGF2α) [41] MDA (malondialdehyde) [10,24,28,29,42]
Oxidized DNA metabolites	8-hydroxy-2-deoxyguanosine (8-OH-dG) [21]
Biomarkers associated with protein oxidation	AOPP (advanced oxidation protein products) [43] Oxidized protein hydroperoxides [44] Protein oxidation index [10]
Biomarkers of thiol–disulfide balance	Biomarkers of thiol–disulfide bond balance [14] Protein sulfhydryl (-SH) groups [24]
Biomarkers characterizing the overall oxidative level or the antioxidant capacity in the body	TOS (total oxidant status) [14] D-ROMs (derivatives of reactive oxygen metabolites) [45] TAOC (total antioxidant capacity) [6] PAT (plasma antioxidant capacity) [45] OSI (oxidative stress index) [14]
Reactive nitrogen species	Nitrites (NO_2_-) Nitrates (NO_3_-) Total nitric oxide metabolites [6]
Activity of enzymatic antioxidants	Superoxide dismutase (SOD) Glutathione peroxidase (GPx) Glutathione reductase (GR) Glutathione S transferase (GST) Catalase (CAT) [10,24,29]
Amount of non-enzymatic antioxidants	Reduced glutathione (GSH) [10]
Other biomarkers	Mitochondrial DNA (mtDNA) [46]
	Ratio of mitochondrial DNA (mtDNA) to nuclear DNA (nDNA) [46] Ceruloplasmin [24]

**Table 2:** Potential benefit of oxidative stress biomarkers in the clinical evaluation of asthma.

Biomarker and Source of Finding	Potential Benefit for Assessing Oxidative Stress in Asthma
MDA (in urine, blood, exhaled breath condensate) ↑	→ indicate a systemic OS process [10,24,28,29]
MDA (in nasal secretion *) ↑	→ assessment of condition worsening related to particulate matter and ozone [42]
MDA ↑, SOD ↑ and GPx ↓	→ poorly controlled asthma predictors [10]
8-iso-PGF2α (in blood and sputum) ↑	→ indicate a systemic OS process [41]
8-OH-dG (in urine) ↑	→ indicate a systemic OS process [21]
D-ROMs (in blood), TOS (in blood and exhaled breath condensate) ↑	→ indicate the overall oxidative status [47]
PAT (in blood) ↑	→ indicate the overall antioxidant capacity [45]
TAOC (in blood and exhaled breath condensate) ↓	→ indicate the overall antioxidant capacity, assess asthma control [6]
OSI (mathematically derived value) ↑	→ indicate the oxidative stress index [14]
Nitrites, nitrates, total nitric oxide metabolites ↑	→ sign of uncontrolled asthma [6]
Thiol disulfide bond balance (in blood) ↑	→ indicate a systemic OS process [14]
Protein-SH groups ↑	→ assess the state of the antioxidant system [24]
Amino acid and protein hydroperoxides (in blood) ↑	→ sign of airway inflammation [44]
Total protein oxidation index and AOPP (in blood) ↑	→ asthma control assessment [10,43]
MtDNA ↑	→ specification of Th2-independent severe asthma endotype [46]
MtDNA/nDNA (in blood, exhaled breath condensate) ↑	→ assessment of asthma severity [46]

↑—increase, ↓—decrease, MDA—malondialdehyde, OS—oxidative stress, SOD—superoxide dismutase, GPx—glutathione peroxydase, 8-iso-PGF2α—8-iso-prostaglandin F2 alpha, 8-OH-dG—8-hidroxy-2′-deoxyguanosine, D-ROMs—derivatives reactive oxygen metabolites, TOS—Total oxidant status, PAT—plasma antioxidant capacity, TAOC—total antioxidant capacity, OSI—oxidative stress index, AOPPs—advanced oxidation protein products, MtDNA—mitochondrial DNA, nDNA—nuclear DNA. * new biomarkers of oxidative stress.

## Data Availability

No new data were created or analyzed in this study. Data sharing is not applicable to this review article.
